# Development of a rapid dipstick with latex immunochromatographic assay (DLIA) for diagnosis of schistosomiasis japonica

**DOI:** 10.1186/1756-3305-4-157

**Published:** 2011-08-09

**Authors:** Li-Ling Yu, Jian-Zu Ding, Li-Yong Wen, Di Lou, Xiao-Lan Yan, Li-Jun Lin, Shao-Hong Lu, Dan-Dan Lin, Xiao-Nong Zhou

**Affiliations:** 1 Institute of Parasitic Diseases, Zhejiang Academy of Medical Sciences, Hangzhou, 310013, China; 2 Jiangxi Provincial Institute of Parasitic Diseases, Nangchang, 330046, China; 3 National Institute of Parasitic Diseases, Chinese Center for Disease Control and Prevention, Shanghai, 200025, China

## Abstract

**Background:**

Schistosomiasis japonica (schistosomiasis) is a zoonosis that can seriously affect human health. At present, the immunodiagnostic assays for schistosomiasis detection are time-consuming and require well-trained personnel and special instruments, which can limit their use in the field. Thus, there is a pressing need for a simple and rapid immunoassay to screen patients on a large scale. In this study, we developed a novel rapid dipstick with latex immunochromatographic assay (DLIA) to detect anti-*Schisaosoma japonicum *antibodies in human serum.

**Results:**

Using latex microspheres as a color probe, DLIA was established to test standard positive and negative sera, in comparison with the classical enzyme-linked immunosorbent assay (ELISA). The sensitivity and specificity of DLIA were 95.10% (97/102) and 94.91% (261/275), respectively. The cross-reaction rates with clonorchiosis, intestinal nematodes, *Angiostrongylus cantonensis *and paragonimiasis were 0, 0, 0 and 42.11% respectively. All the results showed no significant difference to the ELISA. In field tests, 333 human serum samples from an endemic area were tested with DLIA, and compared with ELISA and Kato-Katz method. There was no significant difference between DLIA and ELISA on positive and negative rates of detection; however, significant differences existed between DLIA and Kato-Katz method, and between ELISA and Kato-Katz method. The kappa value between DLIA and ELISA was 0.90.

**Conclusions:**

This is the first study in which DLIA was used to detect anti-*Schistosoma japonicum *antibody. The results show that DLIA is a simple, rapid, convenient, sensitive and specific assay for the diagnosis of schistosomiasis and is therefore very suitable for large-scale field applications and clinical detection.

## Background

Human schistosomiasis is a zoonosis that has endangered human health for thousands of years [1,2]. A recent report indicates that approximately 200 million people are infected and over 750 million people are at risk of infection [3]. Five species of the Schistosomatidae family are human parasites and, in China, the disease is caused by schistosomiasis japonica (schistosomiasis) [2-5]. It has been endemic for centuries and remains a serious public health problem in China [6]. According to the Chinese annual disease report, there are 365 770 patients and approximately 250 million people are at risk in 2009 [7].

The *Schistosoma *infection rate has decreased with improvements in disease control, thus, diagnosis is the key to schistosomiasis control. The diagnostic methods include direct parasitological observations (parasite egg detection and miracidium hatching) and indirect serological techniques (antibodies or circulating antigen detection in serum) [4]. Microscopic examination of stool (e.g., Kato-Katz method and miracidium hatching tests) is considered the "gold standard" for the detection of schistosomiasis [8]. However, these procedures are time-consuming and limited in sensitivity [9-11] due to great day-to-day fluctuations in egg output. Meanwhile, the Kato-Katz method is based on microscopic examination of a 41.7 mg faecal sample, and this quantity is quite small, which can cause high false negative rates. Therefore, the detectable rate is much lower in the Kato-Katz method which depends on the intensity of infection [12]. It had been reported that in a lightly endemic area for schistosomiasis, the disease would not be detected by the Kato-Katz method [13], since the stool sample investigated normally contains less than 2% of the daily egg output [12]. This situation calls for the development of more sensitive methods to supplement or replace stool examination, because less sensitive diagnostic assays are unsuitable for the evaluation of control programmes, such as morbidity reduction by chemotherapy, which normally leads to a reduction in prevalence and an increase in the number of low intensity infections.

Immunodiagnostic assays are already used in China for selective chemotherapy and for the large-scale screening of target populations. Over the past 10-20 years, the most promising alternative diagnostic methods developed against schistosomiasis are the intradermal test (ID) [14], the circumoval precipitin test (COPT) [15], an indirect hemagglutination assay (IHA) [16], and enzyme-linked immunosorbent assay (ELISA) [17]. These methods have high sensitivity and specificity and have been widely used in China. Nonetheless, these methods have some shortcomings, such as being time- consuming and requiring special equipment and reagents, which make them unfit for field and bed application. Thus, better and more convenient screening methods are urgently needed for the detection of schistosomiasis.

Immunochromatography assay is a simple, rapid and convenient method. As a type of new diagnostic technology, it was developed after the immunofluorescence assay, ELISA and radioimmunology assay. An immunochromatographic assay was first used during the early 1990s to test for human chorionic gonadotropin (HCG) [18] and then subsequently for the detection of hepatitis B virus surface antigen (HBsAg) [19]. Now, it is widely used in various fields of medicine. In this study, we used latex microspheres as a color probe to establish a novel, simple, rapid and convenient immunoassay with high sensitivity and specificity for the screening of *Schistosoma *infected persons on a large scale in endemic areas and assess its usefulness for clinical diagnosis. On the basis of the immunochromatographic assay, we labelled mouse anti-human IgG with latex microspheres, and developed a rapid anti-*Schistosoma *antibody detection dipstick, which we named the dipstick with latex immunochromatography assay (DLIA). Its application has not been reported previously elsewhere. We assessed the diagnostic value of the DLIA in the laboratory and field for detection of anti- *Schistosoma *antibodies, and compared the results with the ELISA and Kato-Katz method.

## Material and methods

### Soluble egg antigen (SEA) of *Schistosoma japonicum*

The ova of *Schistosoma *were collected from liver tissue of artificially infected rabbits. After repeated washing, the purified ova were ground into a fine powder using a pestle and mortar. Subsequently, this powder was diluted with normal saline to make a 1% SEA solution. After alternate cryorupture at temperatures of -20°C and 37°C for three rounds, the solution was kept for three days at 4°C. The solution was ultrasonicated on ice three times (2 min each time). After 30 min centrifugation at 851 × g followed by, 20 min at 7661 × g, a clear liquid with a protein content of 15 mg/ml resulted and was stored at -20°C.

### Antibodies for labelling and quality control

Mouse anti-human IgG was obtained from Shanghai Biology Institute, China, the protein concentration was 5 mg/ml and the ELISA titer was 1:50,000. Goat anti-mouse IgG was obtained from Shanghai Biology Institute, China, the protein concentration was 12 mg/ml and its agar gel precipitation titer was 1:64. HRP enzyme-labelled goat anti human IgG was produced by Hangzhou Longji Biotechnology Co. Ltd, China.

### Procedure of mouse anti -human IgG labelled with latex microspheres

Red latex particles and borax buffer (pH 8.5) were mixed and centrifuged for three minutes at 19 000 ×g. The liquid supernatant was discarded, meanwhile 4-morpholineethanesu (MES), N-hydroxysuccinimide (NHS) and 1-ethyl-3-(3-dimethyllaminopropyl) carbodiimide hydrochloride (EDC HCL) was added into the precipitate. After 30 min, the solution was centrifuged for 30 min at 19,000 ×g before the liquid supernatant was discarded again. Next, the precipitate was dissolved in borax buffer (pH 8.5), added mouse anti-human IgG into the solution, and shaking for 30 min and then centrifuged for 30 min at 19 000 ×g. The liquid supernatant was discarded, and the precipitate was blocked with casein blocking buffer. The final concentration of mouse anti-human IgG was 1 mg/ml. The color probe was kept at 4°C. All chemicals were purchased from Hangzhou Longji Biotechnology Co. Ltd, China.

### DLIA dipstick preparation

**Figure 1:**
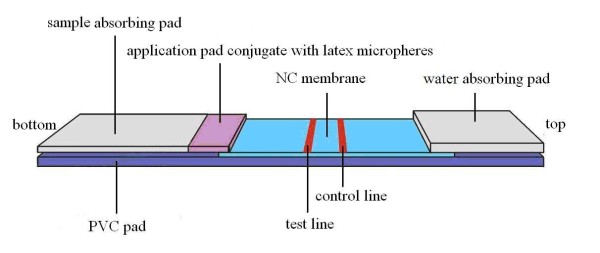
**Schematic diagram of the DLIA dipstick**.

The dipsticks were assembled on a PVC pad (40 mm×300 mm). The dipstick consisted of a water-absorbing pad, an application pad, a sample adsorbing pad and an NC membrane. On the top of the PVC pad, a paper pad (Biotechnology Shanghai Co. Ltd., China) was attached as the water-absorbing pad. At the bottom of the dipstick, glass fibers (Biotechnology Shanghai Co. Ltd) were attached as the sample absorbing pad. The application pad was attached next to the sample pad, which was made of glass fiber membrane (Biotechnology Shanghai Co. Ltd) for conjugated latex microspheres (200 nm, red; Biotechnology Shanghai Co. Ltd) labelled with mouse anti-human IgG. The NC membrane (Millipore SHF 135, USA) was attached between the application pad and the water-absorbing pad and was used as an adsorption pad. SEA was attached on the test line and goat anti-mouse IgG was attached on the control line. The SEA and goat anti-mouse IgG were jet-positioned on to the NC membrane pad at a distance of 4 mm. After all the pads were assembled on the PVC backing, the combined strips were cut to dipsticks measuring 4 mm×40 mm. The schematic of dipstick is shown in Figure 1.

The optimal conditions for the DLIA dipstick assembly adjustment including the conditions as follows: the concentration and the volume of SEA on the test line, the concentration and the volume of goat anti-mouse IgG on the control line, the dilution proportion of latex conjugate, the volume of serum used for testing, reaction time, and so on. A first step of the process of adjustment, involved several different concentration of SEA (such as 0.04, 0.08, 0.16, 0.32 mg/ml respectively) sprayed on the test line, meanwhile, the other conditions of the dipstick was under the assumption value. Next, the dipstick was assembled and tested with the positive and negative standard serum respectively. Then, we compared the sensitivity, specificity and dipstick background, and chose the best concentration of SEA. The others conditions were tested in a similar manner, and finally, we obtained the optimal conditions (data not shown).

### DLIA procedure

**Figure 2:**
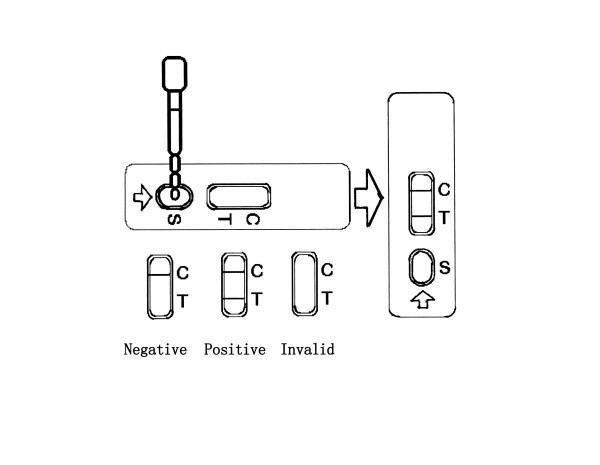
**Dipstick with latex particle immunoassay for the detection of schistosomiasis**.

Analysis in sera samples was done by adding 50 μl serum on to the sample absorbing pad well. The reaction of the anti-SEA antibody and mouse anti-human IgG labelled with latex microspheres took place immediately at the conjugate pad area. Then, the compound of anti-SEA antibody with latex colour probe was combined with SEA which sprayed on the test line if there was anti-SEA in the serum. If anti-SEA was absent, no reaction would occur. The rest of the conjugate then passed though the test line and bind with goat anti-mouse IgG at the control line. The intensity of color developed due to the presence of tracer and this correlate with the amount of anti-SEA antibody present in the sample. If the test line and control line were colored red, the sample was recorded as positive. If only the control lined was colored red but the test line was not colored, the test result was negative. If neither of the lines on the dipstick were colored, the test reagents were assumed not to be working and the test had to be repeated. The result of dipstick latex particle immunoassay for schistosomiasis detection is shown in Figure 2.

In all these experiments, the results must be read at 15 min after the sample added. Some development on the test line was noted after 15 min, and the trace of any line should be held void.

### Serum samples

102 standard positive samples for schistosomiasis were parasite positive by the Kato-Katz method [eggs per gram (EPG): 8~1352]. In total, 275 standard negative samples for schistosomiasis were obtained from healthy people. The 15 serum samples with clonorchiosis, 19 serum samples with paragonimiasis, 11 serum samples with intestinal nemathelminthiasis, 8 serum samples with *Angiostrongylus cantonensis*(all sera provided by Zhejiang Academy of Medical Sciences, China). The sera were stored in aliquots at -20°C.

### Field application

A village in a *Schistosoma*-endemic area (Jiangxi Province, China) was selected as a study site. The 333 persons from 6-65 years old in the village were randomly collected to offered serum samples and stool samples. All the serum samples were tested by DLIA and ELISA respectively. And all the faecal samples were tested only by Kato-Katz method.

### ELISA

SEA antigen (120 μl, 10 μg/ml) was added into the ELISA plates and incubated at 4°C overnight. The plate was washed with phosphate-buffered saline (PBS) before the microwells were blocked with blocking solution. Then, 100 μl of diluted human serum (1:200) was added and the microwells incubated at 37°C for 30 min. The plate was washed again and 100 μl of horseradish peroxidase -labelled goat anti-human IgG (1:2000 diluted) was added and incubated at 37°C for 30 min. After washing, the substrate 3,3-,5,5--tetramethyl benzidiny (TMB) was added into each well. The reaction was stopped by adding 50 μl of H_2_SO_4 _(2 mol/l). Absorbance was measured at 450 nm by an ELX800 multilabel plate reader (Biotek, USA).

### Kato-Katz method

All the faecal samples were tested only by Kato-Katz method. The procedure of the Kato-Katz method for detection of *Schistosoma *eggs was conducted as previously described [13]. Stool samples were processed following the procedure of Kato-Katz method. Three slides, each containing 41.7 mg of sieved stool were made from each stool specimen by qualified technicians and were examined in a blinded manner. The number of eggs in each slide was counted and recorded. The infection intensity of each individual was recorded as EPG (eggs per gram feces) calculated by summing the results of three slides and multiplying by 8.

### Youden index

The Youden Index is used for evaluate the authenticity of the test, which means the capability of the screening test method to distinguish between the real patients and healthy people. The higher the Youden Index of the method, the greater authenticity of the screening test, and the better of the diagnostic kit.

### Stability of DLIA

The DLIA dipsticks were stored at 4°C, 25°C and 37°C, and tested for sensitivity and specificity of the dipsticks by positive and negative standard serum each week.

Three batches of latex conjugated microspheres were synthesized and assembled into dipsticks. All the dipsticks were tested with sensitivity and specificity by positive and negative standard serum.

### Ethical considerations

Written ethical approval for this study was obtained at the Ethics Committee of Zhejiang Academy of Medical Sciences, China.

Written informed consent was obtained from all subjects and from parents or guardians of minors who were involved in the project. Study participants identified as positive for schistosomiasis were treated with 40 mg/kg praziquantel.

### Statistical analysis

The SPSS statistical package for social sciences, version 11.5 software (SPSS Inc., Chicago, IL, 2002) was used for statistical analysis. In the field application of DLIA, only data from individuals who had provided stool specimen were included in the final analysis.

## Results

### Development of DLIA

DLIA was established using SEA as the target antigen on the test line, with goat anti-mouse IgG as the second antibody on the control line. Red latex labelled with mouse anti-human IgG was used as the color probe. To compare the sensitivity, specificity and dipstick background, the optimal conditions for the DLIA dipstick assembly were determined to be as follows: (1) the concentration of SEA was 0.16 mg/ml and the volume of application was 1.0 μl/cm; (2) the concentration of mouse anti-human IgG was 2.0 mg/ml and the volume application was 1.0 μl/cm; (3) the dilution proportion of latex conjugate was 1:6 and the volume application was 2.0 μl/cm; (4) the volume of serum used for testing was 50 μl; and (5) the test result was judged after 15 min by direct observation. The schematic of dipstick is shown in Figure 1.

### Sensitivity

102 serum samples that had been shown to be positive for the Schistosomiasis parasite by the Kato-Katz method (EPG: 8~1352) were used to test the sensitivity of DLIA. ELISA was also performed on these samples for additional comparison. The sensitivity of DLIA and ELISA were 95.10% (97/102) (Table 1). There was no difference in sensitivity between these two assays.

**Table 1:** Serum detection of schistosomiasis by DLIA and ELISA

Group	Number of sera	DLIA		ELISA	
			
		Number positive	Positive detection rate (%)	Number positive	Positive detection rate (%)
Schistosomiasis	102	97	95.10	97	95.10
Normal individuals	275	14	5.09	7	2.55
Clonorchiosis	15	0	0	0	0
Paragonimiasis	19	8	42.11	9	47.37
Intestinal nematodes	11	0	0	0	0
Angiostrongylus cantonensis	8	0	0	0	0

### Specificity

Overall, 275 serum samples from healthy individuals (i.e., negative for schistosomiasis on the Kato-Katz method) were used to assess the specificity of DLIA and ELISA. The specificity of DLIA and ELISA were 94.91% (261/275) and 97.45% (268/275), respectively (Table 1). There was no significant difference in specificity between the two assays (χ^2 ^= 2.43, *P *> 0.05).

### Youden index

Youden index of DLIA and ELISA were 0.90 and 0.93 for schistosomiasis diagnosis, respectively.

### Cross-reactivity

Fifteen clonorchiosis, 11 intestinal nematode and 8 *Angiostrongylus cantonensis *samples were tested with DLIA and ELISA, and showed no signs of cross-reactivity (Table 1). When 19 paragonimiasis samples were tested by DLIA and ELISA, the cross reaction rates were 42.11% (8/19) and 47.37% (9/19), respectively (Table 1), but there was no significant difference in the cross-reactivity between the two assays (χ^2 ^= 0.11, *P *> 0.05).

### Stability of DLIA

The DLIA dipstick was stored for 1 year at various temperatures (i.e., 4°C, 25°C and 37°C) without loss of activity (data not shown). Reconstituted detection reagent could be stored at 4°C, 25°C, and 37°C for at least 1 year. Three batches of latex conjugated microspheres were tested after assembly into dipsticks and these results showed that the dipsticks were stable in each batch (data not shown).

### Field application of DLIA

Serum samples (333 in total) from local people in an area endemic for schistosomiasis were tested by DLIA and ELISA. Fecal samples from these people were only tested by the Kato-Katz method. The positive rates by DLIA and ELISA were 21.62% (72/333) and 24.02% (80/333), respectively. There was no significant difference in positive detection rate between DLIA and ELISA (χ^2 ^= 0.55, *P *> 0.05). Seventy samples were positive and 251 were negative in both the DLIA and ELISA tests. Two cases tested positive by DLIA but were negative by ELISA, and 10 cases that tested positive by ELISA were negative by DLIA. The DLIA results are compared with ELISA, there was 96.40% agreement (321/333). The kappa value between DLIA and ELISA method was 0.90, which indicated that the observed value of DLIA had mid-range coincidence with ELISA (Table 2).

**Table 2:** Detection of schistosomiasis during the field application of DLIA and ELISA

DLIA	ELISA		Total number of samples
		
	Number positive	Number negative	
Number positive	70	2	72
Number negative	10	251	261
Total	80	253	333

Stool examination conducted for all 333 persons by Kato-Katz method gave a positive rate of 3.90% (13/333). There was significant difference in positive rate between DLIA and Kato-Katz method (χ^2 ^= 46.94, p < 0.05). Both assays recorded positive detection in 12 samples, while the tests were both negative in another 260 samples. A further 60 cases tested positive by DLIA but negative by Kato-Katz method, and only one case tested positive by Kato-Katz method but was negative in the DLIA Assay (Table 3).

**Table 3:** Detection of schistosomiasis during the field application of DLIA and the Kato-Katz method

DLIA	Kato-Katz		Total
		
	Number positive	Number negative	
Number positive	12	60	72
Number negative	1	260	261
Total	13	320	333

## Discussion

In this study, DLIA was used for the detection of anti-*Schistisoma *antibodies in human sera at first time and the results showed a 95.10% (97/102) sensitivity and a 94.91% (261/275) specificity for detection. The cross-reaction rates with clonorchiosis, intestinal nematodes, *Angiostrongylus cantonensis *and paragonimiasis were 0 (0/15), 0 (0/11), 0 (0/8) and 42.11% (8/19), respectively. Compared with ELISA (the classic diagnosis method), the results showed that no significant differences existed between the two methods. Youden index of DLIA and ELISA were 0.90 and 0.93 for schistosomiasis diagnosis, respectively.

Importantly, in addition to being rapid and simple to use, the new DLIA technique does not require any specific instruments and can be stored at 4°C, 25°C and 37°C for at least one year. Besides this, the DLIA dipstick could be easily delivered anywhere, so, it is very suitable for rapid diagnosis and large scale applications in the field.

Currently, a few immunochromatographic assay technologies have been used for the diagnosis of schistosomiasis japonica, such as the dipstick dye immunoassay (DDIA) [20] and the colloidal gold immunochromatographic assay (GICA) [21]. DIGFA as a rapid serological test method was based on immunofiltration assay and applied in China for several years. DIGFA and GICA use colloidal gold as the color probe, while DDIA uses a colloidal dye as the color probe. Compared with these kits, DLIA has higher sensitivity and specificity than GICA [21], a lower cross-reaction rate than DDIA (42% compared with 70% for DDIA) [20], and is more convenient to use than DIGFA. The DLIA uses latex microspheres as color probes. Latex microspheres are a type of polymer that binds to particles and disperses throughout a fluid but poses no threat as an environmental pollutant. The carboxyl group of the latex microspheres is activated and binds to the amino-group on the protein. This chemical bond is stable and is not affected by changes in pH, salinity and ionic strength of the environment, which makes it convenient to store for a long time. As a marking agent, the diameter of latex particles is 200 nm, which means results will show up clearly with very tiny amounts of antigen or antibody-label bound to it. In contrast, the diameter of colloidal gold is much smaller than latex particles that means much more antigen or antibody is needed during the labelling step otherwise the result on the nitrocellulose (NC) membrane will not be clear enough to read. The test area on the dipstick used colloidal gold as color probe which could show more clearly with either a higher concentration of antigen or antibody, or with a larger diameter of immunogold (e.g., 40~50 nm). However, although larger colloidal gold increases the sensitivity of the test, the colloidal gold conjugate would be less stable and would precipitate easily. Moreover, the process of immunogold probe labelling would be affected by changes in pH, salinity and ionic strength of the milieu. Furthermore, due to the physical combination between immunogold and protein [22], it is easily dissociated by changes in pH, salinity and ionic strength. So, latex microspheres have several advantages as a color probe compared with colloidal gold. So, as the results in this study proved, the dipstick based on latex microspheres has a higher sensitivity than that based on colloidal gold. As a color probe, latex microspheres can be kept at 4°C -37°C for at least 1 year, which is much longer than that of colloidal gold and colloidal dye [20,21]. Despite the cross-reaction rate of paragonimiasis by DLIA was 42%, which is much lower than the 70% cross-reactivity of DDIA [20], this is still a concern with the dipstick method. Reducing this cross-reactivity will be addressed in further studies.

As a classic method, ELISA is currently the most sensitive method for schistosomiasis diagnosis [23]. As a rapid and convenient schistosomiasis screening test method, DLIA was used in a field test on 333 human sera samples and compared with ELISA. The results of the tests showed that there were no significant differences in positive and negative detection rates between the two methods. According to Feiss's theory, a kappa value of 0.75~1.00 means well-matched, 0.4~0.74 means a distinct correlation and 0.01~0.39 means no match. Thus, the kappa value of field application was 0.90, which means that DLIA has the same ability as ELISA for identifying patients with or without schistosomiasis. However, ELISA reagents require cold-chain logistics and the test takes several hours to complete. Another advantage of DLIA is cost, as the latex microspheres produced in China are considerably less expensive than enzyme conjugates or colloidal gold. In addition, the control band on each dipstick contributes to the quality control of the assay.

However, microscopic examination of stool (e.g., Kato-Katz method and miracidium hatching tests) is still considered the "gold standard" for the detection of schistosomiasis. In this research, the positive rate of stool examination was much lower than DLIA and ELISA. This could be explained by one or more of the following reasons. Firstly, the false negative rate of Kato-Katz method is very high in light epidemic areas. The low sensitivity ranging from 20% to 70% for diagnosis of *Schistosoma *infection [24-26]. Previous research showed that 50% of the patients in a schistosomiasis endemic area where infection rate is less than 25% *Schistosoma *fail to be detected by Kato-Katz method [17]. Furthermore, as the infection rate of schistosomiasis becomes lower [27], it is more and more difficult to identify patients correctly by the Kato-Katz method. Secondly, a recent infection may not have allowed sufficient time after chemotherapy for the antibody level to decline to a normal level, as the anti-*Schistosoma *antibody in the serum would take months or even years to normal level after treatment [28-31]. And because of it, at present, all the serologic diagnosis methods for schistosomiasis which used to observe the success of treatment were only conducted in the laboratory, but not practiced in the field. Until this time, it is not possible to expect that the existing immunodiagnostic approach can meet the requirements for control programs of schistosomiasis and the DLIA, like the other serologic diagnosis methods, was unfit for observe the success of treatment. Thirdly, cross-reactions with other parasitic infections can cause false positive results. Notably, He et al. reported that it was not entirely correct to judge the reliability of immunodiagnostic methods by the results of stool examination [32].

According to the results of the test, the DLIA dipstick could be stored at4°C~37°C for at least one year, and the test could be finished in 15 min without any instrument or reagents. So, it could be easily delivered anywhere, and very suitable for rapid diagnosis and large- scale applications in the field.

## Conclusions

The present study is the first report to use latex microspheres as a color probe to test for schistosomiasis. The results of the research showed that the DLIA assay has the same ability as ELISA to identify schistosomiasis patients and to show that the DLIA has high sensitivity and specificity with no cross-reaction with clonorchiosis, intestinal nematodes and *Angiostrongylus cantonensis*. In summary, DLIA provides a rapid, simple, convenient, easy to carry and reliable diagnosis for schistosomiasis detection, especially for screening patients in a large- scale or for fast clinical diagnosis in the laboratory. Further studies are required to reduce the cross-reaction rate and focus on developing a semi-quantitative or fully quantitative method. In the future, we also planned to develop a series of dipstick based on the immunochromatography assay to test for paragonimiasis, toxoplasmosis and other infections.

## Competing interests

The authors declare that they have no competing interests.

## Authors' contributions

LY and JD developed the DLIA dipstick, LY, JD and DL contributed to sensitivity, specificity, cross-reaction, LY, DL and XZ contributed to field application detection. LL, XY and SL established the test data bank and analyzed the test results. Liyong Wen created the detailed experimental design. All authors read and approved the final version of the manuscript.
